# Factor VIII *in vitro* bioequivalence of denecimig (Mim8) hemostatic effect by thrombin generation assays

**DOI:** 10.1016/j.rpth.2025.103333

**Published:** 2026-01-02

**Authors:** Jacob Lund, Mirella Ezban, Kasper Jensen, David Lillicrap

**Affiliations:** 1 Rare Blood Disorders, Global Research, Novo Nordisk A/S, Bagsværd, Denmark; 2 Formerly Novo Nordisk A/S, Bagsværd, Denmark; 3 Department of Pathology & Molecular Medicine, Richardson Laboratory, Queen's University, Kingston, Ontario, Canada

**Keywords:** bioequivalence, bispecific antibodies, factor VIII, hemophilia, thrombin

## Abstract

**Background:**

Denecimig (Mim8, Novo Nordisk A/S) is a next-generation bispecific antibody designed to mimic activated factor (F)VIII and restore hemostasis in persons with hemophilia A. The extent to which activated FVIII mimetics, such as denecimig and emicizumab, can correct clotting deficiency remains unclear.

**Objectives:**

To assess the *in vitro* FVIII bioequivalence of denecimig hemostatic activity using thrombin generation assays (TGAs).

**Methods:**

Thrombin generation was analyzed in severe hemophilia A platelet-poor plasma, spiked with various levels of FVIII or with clinical doses of denecimig (5 μg/mL) or emicizumab (50 μg/mL), using a sequence-identical analog (SIA). TGA used 4 trigger conditions: tissue factor (TF), activated FXI, a combination of TF and activated FXI, or activated FIX.

**Results:**

The average FVIII bioequivalence estimate using 1 pM TF trigger was 42 IU/dL (SD, 14) for 5 μg/mL denecimig and 14 IU/dL (SD, 5) for 50 μg/mL emicizumab-SIA using peak thrombin. The FVIII bioequivalence estimates of denecimig and emicizumab-SIA for hemostatic activity were generally highest for endogenous thrombin potential, followed by peak thrombin and velocity index across trigger concentrations. FVIII bioequivalence increased with decreasing trigger concentrations. Denecimig demonstrated a higher thrombin peak in conditions with limited activated FIX compared with emicizumab-SIA, suggesting differences in mechanisms of action.

**Conclusion:**

Estimation of denecimig *in vitro* FVIII bioequivalence with TGA depends on the TGA parameter and the type and concentration of the trigger. Denecimig demonstrated higher *in vitro* FVIII bioequivalence than emicizumab-SIA, indicating the potential for effective hemostatic coverage. Further studies are needed to fully understand the hemostatic effects of bispecific antibodies.

## Essentials

• The degree of clotting correction by activated FVIII mimetics for hemophilia A remains unclear.
• *In vitro* FVIII bioequivalence of denecimig activity was assessed by TGA.
• Denecimig demonstrated higher FVIII bioequivalence than a sequence-identical analog of emicizumab.
• Estimation of FVIII bioequivalence depends on the TGA parameter and the trigger type and concentration.

## 1 Introduction

Hemophilia A is a genetic disorder characterized by a deficiency of factor (F)VIII, leading to impaired blood clotting and a risk of bleeding episodes, particularly in the joints [1]. Traditional treatment options have primarily involved the regular administration of FVIII concentrates, which, while effective, come with challenges such as the development of FVIII inhibitors, the need for frequent dosing, intravenous administration, and the risk of infusion-related complications [2]. New directions in hemophilia therapy development emphasize the need for treatments that offer high efficacy, reduced treatment burden, and improved patient quality of life [2].

One such advancement is targeting higher hemostatic activity, which is associated with greater bleeding prevention [3,4].

Activated FVIII (FVIIIa) mimetics are a new class of nonfactor therapy designed to prevent bleeding and reduce treatment burden through subcutaneous administration in persons with hemophilia A, both with and without FVIII inhibitors [5]. FVIIIa mimetics are bispecific antibodies (BiAbs) that mimic the function of FVIIIa by bridging activated FIX (FIXa) and FX, thereby activating FX and leading to thrombin generation and fibrin clot formation [6]. Examples of FVIIIa mimetics include emicizumab (Roche), denecimig (Mim8, Novo Nordisk), NXT007 (Roche), and Inno8 (Novo Nordisk) [5,[7], [8], [9]].

Emicizumab, a humanized bispecific immunoglobulin G4 antibody and the first approved FVIIIa mimetic, has emerged as a paradigm shift in hemophilia A treatment, with a subcutaneous administration route and less frequent dosing compared with factor replacement therapies [5,6,10,11]. Denecimig is a next-generation, fully human bispecific immunoglobulin G4 antibody under development for subcutaneous prophylaxis of hemophilia A. Denecimig has unique binding sites for FIXa and FX, which are distinct from those of emicizumab. It is optimized for increased FIXa proteolytic activity (by more than 20,000-fold compared with native FIXa), as well as reduced binding affinity to FIX and FX in order to decrease the risk of self-inhibition and binding in solution, while supporting efficient FX activation on the surface of activated platelets through avidity binding [7,12].

In clinical trials, denecimig was administered using a tiered dosing approach, in which each patient received a fixed dose based on body weight range and selected dosing frequency. This regimen has been shown to be well tolerated and efficacious in preventing treated bleeds, with a denecimig plasma concentration of 3.8 to 5.7 μg/mL [13,14] and a half-life of approximately 1 month [15]. The FRONTIER2 phase 3 study demonstrated significantly reduced annualized bleed rates (ABRs) compared with prior clotting factor prophylaxis or on-demand treatment, maintained consistently low ABRs across dosing intervals up to once monthly, and was well tolerated with no major safety concerns [14].

While both FVIIIa and FVIIIa mimetics (such as emicizumab and denecimig) aim to restore hemostasis by bridging FIXa and FX, their mechanisms of action differ. FVIII/FVIIIa has an on/off switch mechanism, in contrast to the constitutively active FVIIIa mimetics [16]. Additionally, unlike FVIIIa mimetics, FVIIIa promotes localization to the phospholipid membrane and has a higher binding affinity for FIXa and FX than FVIIIa mimetics [16]. Furthermore, FVIII replacement therapies result in peak and trough FVIII levels, whereas subcutaneously administered FVIIIa mimetics maintain a steady-state level [13,17].

Clinical studies have demonstrated consistent hemostatic efficacy of FVIIIa mimetics; however, the degree to which these mimetics can correct the clotting defect in persons with hemophilia A remains unclear [18]. Understanding the level of hemostatic coverage provided by FVIIIa mimetics relative to well-established FVIII levels may support treatment decisions, provide insights into their efficacy, and inform the clinical management of patients receiving denecimig prophylaxis [19,20].

Multiple approaches have been used to evaluate the FVIII bioequivalent activity of emicizumab. Previous studies employed different hemophilia models (FVIII-deficient mice supplemented with human FIX and FX, persons with mild or moderate hemophilia, or persons with severe hemophilia with and without inhibitors) and assessed FVIII bioequivalence based on either the bleeding phenotype (including ABR) or the thrombin generation assay (TGA) [[21], [22], [23]]. Based on these studies, the FVIII bioequivalence of emicizumab has been estimated at 9 IU/dL [21], 3 to 27 IU/dL [23], and 10 to 40 IU/dL [22].

### 1.1 Methodological challenges in assessing bioequivalence

The TGA is a global hemostatic test used to assess the hemostatic activity of procoagulant hemostatic agents by providing information on the kinetic and quantitative parameters of thrombin generation. These parameters include lag time, time to peak (ttPeak), peak thrombin, endogenous thrombin potential (ETP), and velocity index (rate of thrombin generation, defined as thrombin peak/[ttPeak – lag time]). The assay is performed on plasma samples, most commonly initiated by an extrinsic pathway trigger (eg, tissue factor [TF]) or, alternatively, by a trigger of the intrinsic pathway (eg, activated FXI [FXIa]) [24].

However, certain challenges still beset the TGA in approximating FVIII equivalence of BiAbs [25]. TGA parameters are derived from the thrombin generation curve and may not necessarily reflect other changes induced by treatment or elements of the disease state, such as inflammation resulting from acute hemarthrosis, thereby leading to oversimplification [[25], [26], [27]]. Additionally, TGA parameters are interdependent, requiring a robust statistical analysis to avoid bias [25]. Another limitation is that the TGA does not fully replicate *in vivo* conditions, including blood cells and blood flow [24,28].

Furthermore, TGAs are not well standardized, with divergent methodologies across laboratories at different stages of pretest, test performance, and test interpretation, including blood collection, sample processing, reference ranges, and data analysis [29]. While animal models provide insights into the *in vivo* activity of BiAbs, they do not fully replicate the conditions of persons with hemophilia due to species-specific differences in coagulation factors and regulatory mechanisms [21]. Additionally, pharmacokinetic and pharmacodynamic profiles of BiAbs can differ significantly between animals and humans [21]. The FVIIIa mimetic BiAbs developed to date for clinical use primarily bind human FIXa and FX, thereby necessitating supplementation of these factors to assess *in vivo* activity, adding to the complexity of obtaining translatable findings [21]. Therefore, the results of FVIII bioequivalence obtained from animal models should be evaluated with caution.

Owing to these shortcomings, the TGA was selected to assess the FVIII bioequivalence of denecimig. To account for the high dependence of TGA results on trigger type and concentration [30], this study employed a comprehensive approach, testing multiple triggers at varying concentrations and assessing multiple TGA parameters.

### 1.2 Objective

The objective of this study was to assess the *in vitro* FVIII bioequivalence of the denecimig hemostatic effect using the TGA.

## 2 Methods

### 2.1 Human material

A congenital severe hemophilia A platelet-poor plasma pool (FVIII level < 1%) was obtained from George King Bio-Medical Inc. Levels of von Willebrand factor were normal in the plasma pool.

### 2.2 Reagents

Denecimig and FVIII (turoctocog alfa, B-domain truncated recombinant human FVIII) were produced by Novo Nordisk A/S (Denmark) as previously described [12,31]. A published sequence of emicizumab in the World Health Organization Drug Information was used to generate a sequence-identical analog (SIA) of emicizumab (Roche), which has previously been shown to be comparable to emicizumab in the TGA [12].

A concentration of 5 μg/mL denecimig was selected to reflect its anticipated therapeutic level; this level fell within the plasma concentration range of 3.8 to 5.7 μg/mL, as observed in the FRONTIER2 phase 3 study [14]. The concentration of 50 μg/mL emicizumab-SIA was selected to reflect the trough plasma concentration observed for emicizumab in the HAVEN 1 phase 3 study [32].

Relipidated TF reagent (PPP-Reagent LOW, PPP-Reagent, and PPP-Reagent HIGH; Thrombinoscope BV) and phospholipid vesicles (MP-reagent; Thrombinoscope BV) were obtained from Diagnostica Stago. Human FXIa and FIXa were obtained from Prolytix and diluted in MP-reagent.

### 2.3 Thrombin generation in human plasma

TGA was conducted using the calibrated automated thrombin generation method as previously described [33]. The assay used a severe congenital human hemophilia A platelet-poor plasma pool (platelet count < 10,000/μL), and the triggers were either FXIa or FIXa supplemented with 4 μM phospholipid vesicles (MP-reagent), relipidated TF in MP-reagent, or a combination of TF and FXIa in MP-reagent. Trigger concentrations were 0.5 to 20 pM TF, 0.1 to 8 mU/mL FXIa (either on its own or in combination with 1 pM TF), or 0.39 to 400 pM FIXa.

The thrombin calibrator was from Diagnostica Stago, and thrombin generation was measured at 37 °C using a Fluoroskan Microplate Fluorometer (Thermo Fisher Scientific) with software provided by Diagnostica Stago.

All samples were spiked with the test compound at a 1:10 ratio. For baseline controls and calibration, a buffer was used instead of the test compound to ensure similar dilution of the plasma samples and controls. Stated concentrations of single factors and test compounds refer to their levels in the plasma samples before any dilution or mixing in the assay. Trigger concentrations refer to the final levels in the assay mixture after all dilutions and additions have been made.

### 2.4 Statistical analysis

The fluorescence signal was corrected for α_2_-macroglobulin-bound thrombin activity and converted to thrombin concentration using a calibrator (Thrombinoscope BV) and Thrombinoscope software version 5.0.0.742 (Synapse BV). The following parameters were extracted: lag time (defined as time to 16.7% of peak thrombin), thrombin peak (defined as the maximal thrombin generation in nM), ttPeak, velocity index, and ETP, corresponding to the area under the thrombin generation curve [25,34,35]. The assay range was defined as FVIII levels at which recovery was between 75% and 125% of the spiked level (Supplementary Figure).

Data from samples with varying FVIII concentration curves were fitted using a variable-slope 3-parameter dose-response model (equation [Eq.] 1).

(1) $${\text{TGA}}_{\text{parameter}}=\text{Bottom}+\frac{{\left[\text{FVIII}\right]}_{\text{plasma}}\times \left(\text{Top}-\text{Bottom}\right)}{{\text{EC}}_{50}+{\left[\text{FVIII}\right]}_{\text{plasma}}}$$

where *TGA*_*parameter*_ was either lag time, ttPeak, thrombin peak, velocity index, or ETP; *Bottom* and *Top* were the plateaus observed in the dose-response curve; *[FVIII]*_*plasma*_ was the FVIII plasma concentration in the assay; and *EC*_*50*_ was the FVIII plasma concentration generating half of the maximal signal in the recorded *TGA*_*parameter*_.

Following the fit of the FVIII concentration-response curves for each assay condition and TGA parameter, the FVIII equivalents were estimated from the measured TGA parameters of samples containing FVIII, denecimig, or emicizumab-SIA using Eq. 2 (Eq. 1 solved for [FVIII]_plasma_):

(2) $${\text{FVIII}}_{\text{eq},\text{estimate}}=\frac{{\text{EC}}_{50}\times \left({\text{TGA}}_{\text{parameter}}-\text{Bottom}\right)}{\text{Top}-{\text{TGA}}_{\text{parameter}}}$$

where *FVIII*_*eq,estimate*_ is the estimated FVIII equivalence based on a particular TGA_parameter_. TGA results below EC_5_ or above EC_95_ were excluded from the FVIII equivalence estimate owing to high uncertainty of the estimate.

The assay range was defined as FVIII levels at which recovery was between 75% and 125% of the spiked level.

### 2.5 Ethics statement

The study was conducted in line with the principles of the Declaration of Helsinki. Specimens sourced from George King Bio-Medical Inc., donors provide informed consent at the time of blood collection as part of the supplier’s routine procedures.

## 3 Results

### 3.1 Mechanism of action differences between FVIII and denecimig in relation to TGA

**Figure 1:**
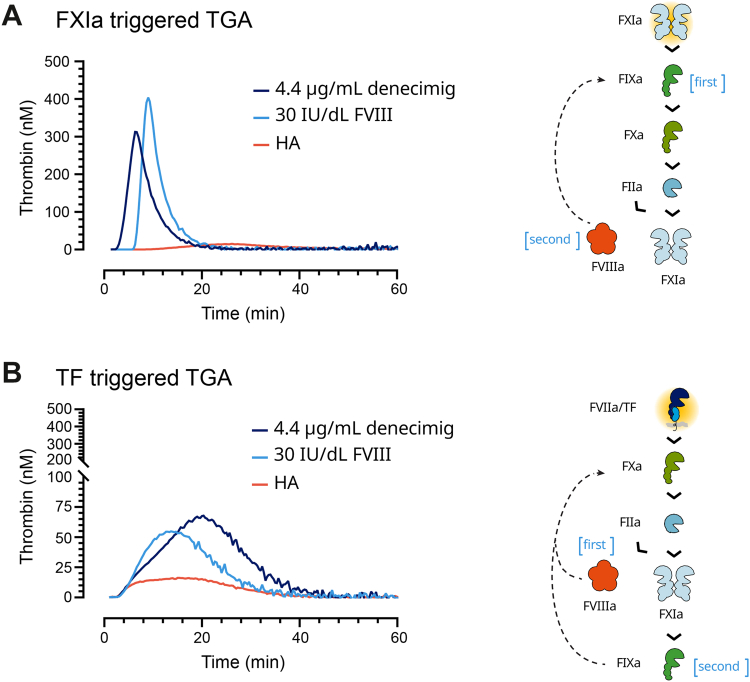
**Differences in mechanisms of action between factor (F)VIII and denecimig in relation to the thrombin generation assay (TGA).** TGA thrombogram in hemophilia A (HA) plasma alone or supplemented with denecimig or FVIII initiated with the triggers (A) activated FXI (FXIa; 8 mU/mL) or (B) tissue factor (TF; 1 pM). The right panels show the kinetic hypothesis of trigger-specific thrombin generation. FVIII and denecimig mode of action in a TGA initiated with FXIa and TF. “First” and “second” indicate sequential steps for each condition. FVIII activation is hypothesized to be the rate-limiting step for TGA triggered with FXIa, whereas activated FIX (FIXa) activation is the rate-limiting step for TGA triggered with TF. A denecimig concentration of 4.4 μg/mL was selected from the trough concentration range in the FRONTIER2 study (4.1 [95% CI, 3.83; 4.56] to 4.5 [95% CI, 4.23; 4.89] μg/mL) [14]. A FVIII concentration of 30 International Units (IU)/dL was selected because it produces a similar peak height and endogenous thrombin potential (area under the curve) for the 2 trigger conditions (FXIa and TF). Furthermore, it has been shown that denecimig had an additive effect on these parameters when the FVIII concentration was ≤30 IU/dL [34]. FIIa, thrombin; FVIIa, activated factor VII; FVIIIa, activated factor VIII; FXa, activated factor.

When triggering thrombin generation with FXIa, denecimig demonstrated a shorter lag time and ttPeak than FVIII, whereas FVIII showed a higher velocity index and thrombin peak than denecimig. When TF was used as a trigger, lag time was comparable between denecimig and FVIII (Figure 1) [34].

These results illustrate the distinct rate-limiting steps in the TGA pathway depending on the trigger. It is hypothesized that FVIII activation is the rate-limiting step for thrombin generation when FXIa is used as the trigger (Figure 1A), whereas FIX activation is the rate-limiting step when TF is used as the trigger (Figure 1B). Due to differences in the rate-limiting steps, the time-based parameters (lag time and ttPeak) were considered less suitable TGA parameters for assessing FVIII bioequivalence and were therefore omitted from thrombin generation in the analyses.

### 3.2 Concentration-response relationship of TGA parameters with FVIII

**Figure 2:**
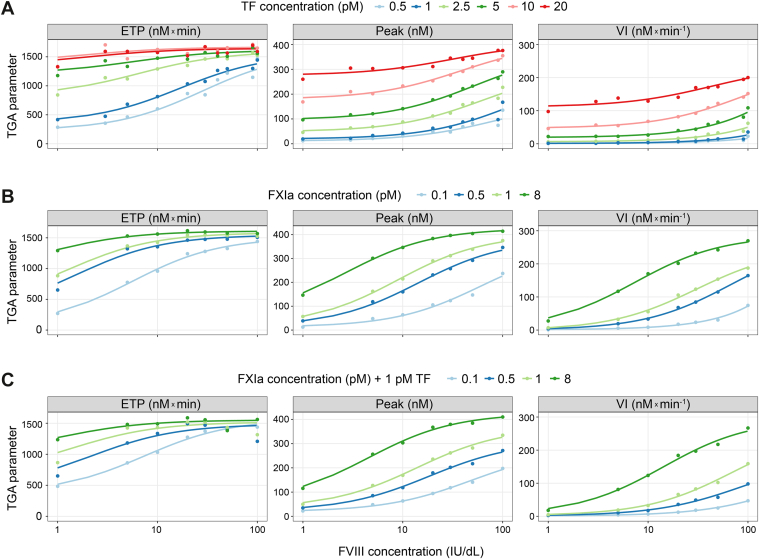
**Concentration response of factor (F)VIII in the thrombin generation assay (TGA) with varying trigger concentrations.** Concentration response of FVIII to different triggers: (A) tissue factor (TF; 0.5-20 pM), (B) activated FXI (FXIa; 0.1-8 milliunits [mU]/mL), and (C) 1 pM TF + FXIa (0.1-8 mU/mL). The x-axis scale is logarithmic (log_10_). Circle markers: FVIII measurements; colored lines: fit to FVIII measurements. ETP, endogenous thrombin potential; IU, International Units; VI, velocity index.

The concentration-response relationship of FVIII in TGAs was evaluated using different trigger conditions and TGA parameters. Increasing the concentration of the triggers (TF, FXIa, and FXIa + 1 pM TF) resulted in greater ETP, thrombin peak, and velocity index with FVIII, demonstrating a concentration-dependent response in thrombin generation (Figure 2). Similar results were obtained with a different synthetic lipid composition and a higher phosphatidylserine/phosphatidylcholine concentration (data not shown).

**Figure 3:**
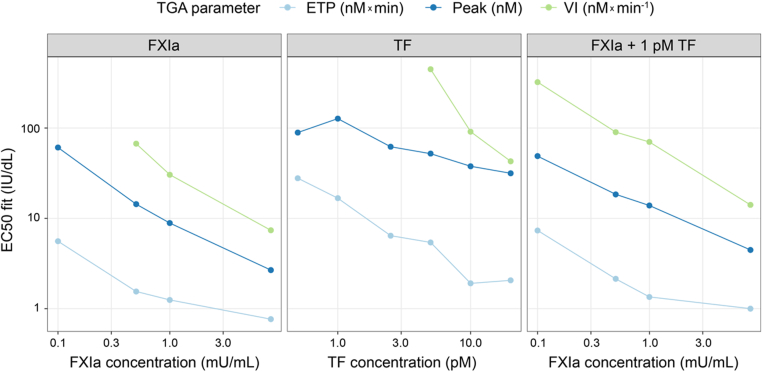
**Factor (F)VIII concentration-response for half maximal effective concentration (EC_50_) estimation**. Fitted EC_50_ values for FVIII concentration in the thrombin generation assay (TGA) response using various trigger concentrations of activated FXI (FXIa), tissue factor (TF), and 1 pM TF + FXIa. Axes scales are logarithmic (log_10_). ETP, endogenous thrombin potential; IU, International Units; mU, milliunits; Peak, thrombin peak; VI, velocity index.

Fitted EC_50_ values for FVIII concentration response in the TGA were assessed as a function of trigger type, trigger concentration, and TGA parameter. Fitted EC_50_ values decreased with increasing trigger concentration and were lowest for ETP, followed by thrombin peak and velocity index (Figure 3). EC_50_ response was dependent on trigger type, trigger concentration, and TGA parameter. At higher trigger concentrations, the FVIII TGA response saturated at a lower FVIII concentration. At lower trigger concentrations, a higher FVIII concentration was required to produce an EC_50_ response, suggesting that FIXa may be the limiting factor (Figure 3).

### 3.3 Concentration-response relationship of TGA parameters with denecimig and FVIII bioequivalence

**Figure 4:**
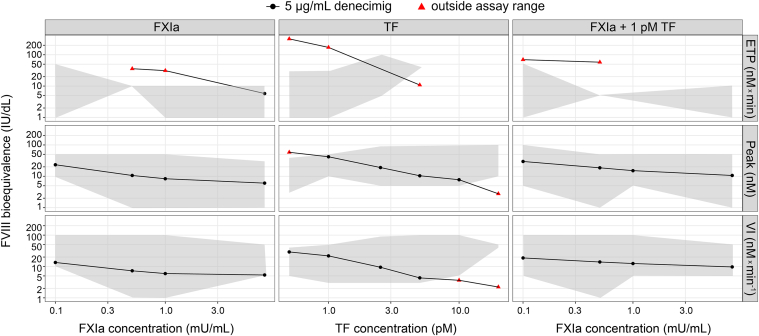
**Assay range for factor (F)VIII bioequivalence of denecimig.** The assay range (gray shaded area) of FVIII concentrations in the thrombin generation assay (TGA) response using various trigger concentrations of tissue factor (TF), activated FXI (FXIa), and 1 pM TF + FXIa. Axes scales are logarithmic (log_10_); gray background: assay range. ETP, endogenous thrombin potential; IU, International Units; mU, milliunits; Peak, thrombin peak; VI, velocity index.

The trigger conditions assessed above with FVIII were applied to samples spiked with denecimig to estimate its FVIII bioequivalence. The assay range was based on a measured recovery of 75% to 125% of the spiked FVIII level. FVIII bioequivalence tended to increase with decreasing trigger concentrations for ETP, thrombin peak, and velocity index (Figure 4). A narrow assay window was observed at high TF trigger concentrations; therefore, these concentrations (above 5 pM) were not included in subsequent analyses. The ETP parameter had a narrow assay window, with most assessed values falling outside the assay range.

**Figure 5:**
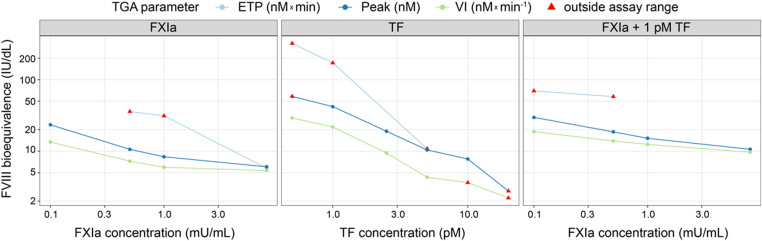
**Effect of thrombin generation assay (TGA) parameters on factor (F)VIII bioequivalence of denecimig under different trigger conditions**. FVIII bioequivalence at 5 μg/mL of denecimig in TGA response using various trigger concentrations of tissue factor (TF), activated FXI (FXIa), and 1 pM TF + FXIa. Axes scales are logarithmic (log_10_). ETP, endogenous thrombin potential; IU, International Units; mU, milliunits; Peak, thrombin peak; VI, velocity index.

The FVIII bioequivalence of denecimig was estimated using different trigger conditions, based on 3 TGA parameters: ETP, thrombin peak, and velocity index. Across all assessed triggers, the FVIII bioequivalence of denecimig was highest for ETP, although most estimations fell outside the established assay range due to a limited assay window, followed by the thrombin peak and velocity index (Figure 5). The TF trigger yielded the highest FVIII bioequivalence for ETP, thrombin peak, and velocity index, which increased at lower trigger concentrations. Using TF as a trigger and at a concentration commonly used for assessing hemophilia plasma samples (1 pM TF) [36], FVIII bioequivalence with 5 μg/mL of denecimig was 42 IU/dL (SD, 14) for the thrombin peak and 22 IU/dL (SD, 9) for the velocity index (Supplementary Table). The ETP estimate was outside the assay range (171 IU/dL [SD, 136]).

### 3.4 Concentration response of FVIII and FVIIIa mimetics with a FIXa trigger

**Figure 6:**
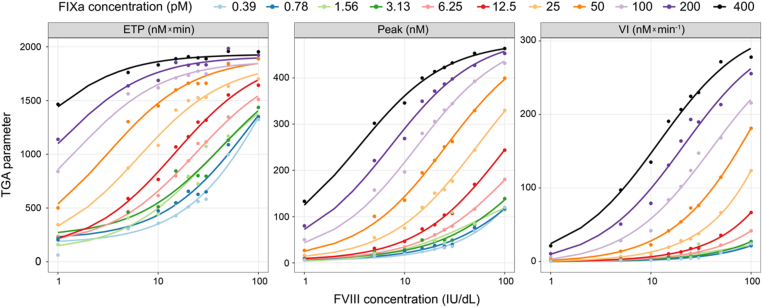
**Effect of activated factor (F)IX (FIXa) trigger concentrations on thrombin generation**. Colored markers: FVIII measurements; colored lines: fit to FVIII measurements. Concentration response of FVIII in the presence of different concentrations of FIXa. The x-axis scale is logarithmic (log_10_). ETP, endogenous thrombin potential; IU, International Units; Peak, thrombin peak; TGA, thrombin generation assay; VI, velocity index.

Based on the concentration-response relationship described above, it was hypothesized that the dependence of FVIII bioequivalence on trigger concentration is driven by a limiting concentration/rate of FIXa generation in the assay. To assess this, varying concentrations of FIXa were used as triggers to determine TGA response to FVIII and FVIIIa mimetics. For FVIII, assay response was highly dependent on the FIXa concentration (Figure 6); higher FVIII concentrations and the FIXa trigger yielded higher TGA responses across ETP, thrombin peak, and velocity index parameters.

**Figure 7:**
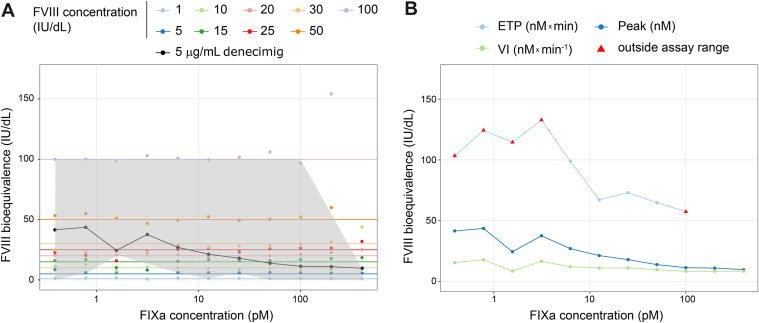
**Factor (F)VIII bioequivalence of denecimig with the activated FIX (FIXa) trigger**. (A) Peak thrombin generation-based FVIII bioequivalence of denecimig with the FIXa trigger. Gray shaded area: assay range; colored markers: FVIII equivalence (FVIIIeq) of FVIII measurements; colored lines: expected FVIIIeq of FVIII measurements; black markers: FVIIIeq of denecimig; black line: predicted FVIIIeq of 5 μg/mL denecimig. (B) Thrombin generation assay parameters for denecimig FVIII bioequivalence with the FIXa trigger. The x-axis scale is logarithmic (log_10_). ETP, endogenous thrombin potential; IU, International Units; Peak, thrombin peak; VI, velocity index.

For denecimig, FVIII bioequivalence increased with decreasing FIXa concentrations (Figure 7A). Estimates of FVIII bioequivalence varied by the TGA parameter. Consistent with previous triggers, FVIII bioequivalence of denecimig using the FIXa trigger was highest for ETP, followed by thrombin peak and velocity index (Figure 7B). ETP showed a limited assay window, with most values falling outside the assay range of the FIXa trigger, suggesting that ETP may be less appropriate for reflecting bioequivalent hemostatic activity.

### 3.5 Estimation of FVIII equivalence of denecimig and emicizumab-SIA

**Figure 8:**
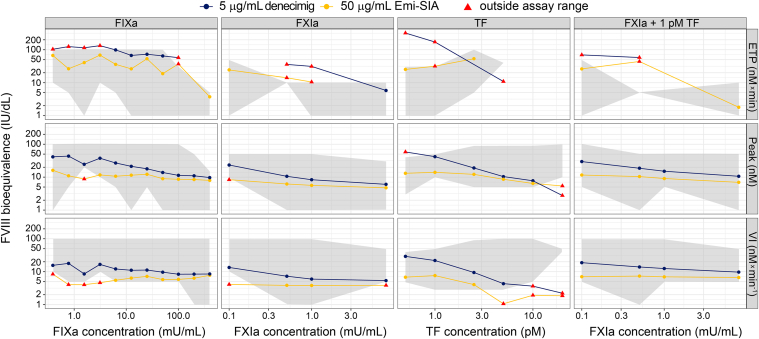
**Factor (F)VIII bioequivalence of denecimig and the emicizumab sequence-identical analog (Emi-SIA).** FVIII bioequivalence of 5 μg/mL denecimig and 50 μg/mL Emi-SIA in the thrombin generation assay response using various trigger concentrations of tissue factor (TF), activated FXI (FXIa), and 1 pM TF + FXI. Axes scales are logarithmic (log_10_). ETP, endogenous thrombin potential; FIXa, activated factor IX; IU, International Units; mU, milliunits; Peak, thrombin peak; VI, velocity index.

FVIII bioequivalence was estimated for denecimig and emicizumab-SIA across all trigger types and at a range of concentrations. For both denecimig and emicizumab-SIA, FVIII bioequivalence was generally highest for ETP, followed by the thrombin peak and velocity index. Across all trigger concentrations and TGA parameters, FVIII bioequivalence of 5 μg/mL denecimig was estimated to be higher than that of 50 μg/mL emicizumab-SIA (Figure 8). The FVIII bioequivalence generally increased with decreasing trigger concentrations.

A higher thrombin peak was observed with denecimig under conditions of limited FIXa compared with emicizumab-SIA. Using 1 pM TF as a trigger, peak thrombin generation values of 85.2 nM (SD, 16.1) for 5 μg/mL denecimig and 46.0 nM (SD, 8.3) for 50 μg/mL emicizumab-SIA were measured, which corresponded to FVIII bioequivalence estimates of 42 IU/dL (SD, 14) for denecimig and 14 IU/dL (SD, 5) for emicizumab-SIA (Supplementary Table). With the same trigger, FVIII bioequivalence for ETP was outside the assay range at 5 μg/mL denecimig (Figure 4) and 50 μg/mL emicizumab-SIA (data not shown). FVIII bioequivalence for velocity index was 22 IU/dL (SD, 9) at 5 μg/mL denecimig and 8 IU/dL (SD, 3) for 50 μg/mL emicizumab-SIA (Supplementary Table), based on velocity index values of 6.7 nM/min (SD, 2.4) for denecimig and 3.1 nM/min (SD, 0.7) for emicizumab-SIA.

## 4 Discussion

In this study, TGA was used to assess the *in vitro* bioequivalence of FVIIIa mimetics in terms of FVIII activity. The estimated bioequivalence was dependent on trigger type, trigger concentration, and FVIIIa mimetic concentration. In the present analysis, FVIII bioequivalence of denecimig and emicizumab-SIA was generally highest for ETP (although often outside the assay range), followed by thrombin peak and velocity index across all triggers and trigger concentrations, as demonstrated by the different thrombin curve shapes; these differences may be attributed to distinct mechanisms of action of FVIII, denecimig, and emicizumab-SIA. Across all trigger concentrations and TGA parameters, the FVIII bioequivalence of denecimig was estimated to be higher than that of emicizumab-SIA; however, the translation of *in vitro* FVIII bioequivalence of FVIIIa mimetics using TGA into clinical efficacy is currently unknown.

The present study explored differences in trigger type and concentration among FVIII, denecimig, and emicizumab-SIA in relation to TGA. When triggering thrombin generation with FXIa, denecimig showed a shorter lag time and ttPeak than FVIII, as demonstrated in previous studies [34]. The differences in lag time and ttPeak between FVIII and FVIIIa mimetics may be attributed to the constitutively active nature of FVIIIa mimetics, whereas endogenous FVIII requires activation by FXa or thrombin before its stimulatory effect can be initiated [34]. As previously hypothesized, FVIII activation is the rate-limiting step for thrombin generation when FXIa is used as the trigger, whereas FIX activation is the rate-limiting step when TF is used as the trigger [34]. Greater thrombin generation was observed when plasma was triggered by FXIa than by TF; this is consistent with a similar study hypothesizing that thrombin forms more quickly with the FXIa trigger due to greater FIXa generation [34].

Independent of the trigger type, the FVIII bioequivalence of denecimig increased with decreasing trigger concentrations for ETP, thrombin peak, and velocity index. This appeared to be more pronounced with denecimig than with emicizumab-SIA and may reflect differences in how the molecules bind and stimulate FIXa activity [12].

Furthermore, denecimig (5 μg/mL) showed a higher thrombin peak in conditions with limited FIXa compared with emicizumab-SIA (50 μg/mL), indicating that denecimig can produce substantial FX activation even in the presence of relatively low FIXa concentrations, potentially reflecting the early phase of coagulation before high levels of FIXa have been generated. Greater thrombin generation with denecimig at lower FIXa concentrations may result from optimization of the anti-FIXa arm of denecimig to enhance the proteolytic activity of FIXa. Denecimig induces conformational stimulation of the FIXa active site, in which FIXa more readily cleaves the scissile bond in FX to generate FXa [12]. In the FRONTIER1 phase 1/2 trial, denecimig generated comparable peak thrombin at 15-fold lower plasma concentrations than emicizumab, using a TGA with a high FXIa trigger concentration [15], illustrating the effect of conditions that generate high FXIa levels. Similarly, in an *in vitro* TGA using platelet-poor and platelet-rich plasma, denecimig significantly improved the thrombin generation capacity of plasma samples from persons with severe hemophilia A and demonstrated a higher thrombin generation capacity compared with emicizumab [37]. The effect of FVIIIa mimetics on *in vitro* thrombin generation and FVIII bioequivalence data does not necessarily translate into clinical efficacy. As such, ongoing research and clinical experience with FVIIIa mimetics are needed to refine the understanding of their hemostatic effects relative to well-characterized FVIII levels, which may help inform treatment decision-making. Identifying the level of hemostatic activity provided by FVIIIa mimetics can help evaluate the risk of breakthrough bleeding and provide reassurance about the risk of thrombotic complications. Additionally, greater thrombin generation with denecimig at lower FIXa concentrations than with emicizumab-SIA is potentially important in the early phase of the hemostatic response and for the treatment of children, whose FIX levels are lower than those of adults [38]. Given the established hemostatic efficacy of first-generation FVIIIa mimetics [39], there remains clinical interest in how next-generation agents may translate enhanced thrombin generation into additional therapeutic benefit.

Clinical studies are necessary to evaluate the efficacy outcomes of FVIIIa mimetics. The current study was performed *in vitro*, which limits its ability to fully replicate the physiological conditions of patients [24,28]. Although the use of platelet-poor plasma is the standard for most coagulation tests, it provides an incomplete representation of hemostasis owing to the key role that platelets play in this process [40,41]. Additionally, individual responses to denecimig can vary with varying levels of other coagulation factors, which were not assessed in this analysis. In addition, fundamental differences in activation mechanisms, potency, and modes of action between FVIII and FVIIIa mimetics preclude complete comparisons [42]. This study identified limitations in using time-based TGA parameters to assess FVIII bioequivalence; however, in certain bleeding situations, it may be clinically informative to assess the time to the thrombin peak of FVIIIa mimetics relative to FVIII.

The current study aimed to comprehensively assess the *in vitro* FVIII bioequivalence of the denecimig hemostatic effect using the TGA. This was achieved through an extensive approach that utilized multiple thrombin generation triggers at varying concentrations, then analyzed the TGA output using various parameters. This detailed assessment might allow for a more complete picture of denecimig activity across different coagulation conditions, potentially informing optimal dosing strategies in different clinical scenarios. Furthermore, analyzing various TGA outputs might provide insights into specific mechanisms of action of denecimig. In the present study, a single concentration was selected to represent therapeutic levels; however, the clinical range varies among patients. Further studies are needed to fully understand the hemostatic effects of BiAbs.

## 5 Conclusion

FVIIIa mimetic BiAbs function as cofactors in the intrinsic tenase complex. Clinical studies have demonstrated robust efficacy in preventing bleeding in persons with hemophilia A with and without inhibitors. However, because these mimetic molecules artificially reconstitute the intrinsic tenase complex, determining their FVIII bioequivalence is challenging. This comprehensive study demonstrated significant variations in determining the hemostatic activity of FVIIIa mimetics using the TGA under different triggers and assay conditions. Nevertheless, denecimig demonstrated 2 consistent features: higher FVIII bioequivalence at lower trigger concentrations and higher *in vitro* FVIII bioequivalence of denecimig compared with emicizumab-SIA at clinically relevant concentrations. Further studies are needed to assess how *in vitro* thrombin generation and FVIII bioequivalence data may translate into clinical efficacy.

## References

[1] Berntorp E., Fischer K., Hart D.P., Mancuso M.E., Stephensen D., Shapiro A.D. (2021). Haemophilia. Nat Rev Dis Primers.

[2] Gogia P., Tarantino M., Schramm W., Aledort L. (2023). New directions to develop therapies for people with hemophilia. Expert Rev Hematol.

[3] Agosti P., Siboni S.M., Scardo S., Torri A., Gualtierotti R., Peyvandi F. (2023). Minimum factor VIII levels to prevent joint bleeding in mild hemophilia A. Blood Adv.

[4] Malec L., Matino D. (2023). Targeting higher factor VIII levels for prophylaxis in haemophilia A: a narrative review. Haemophilia.

[5] Alcedo Andrade P.E., Mannucci P.M., Kessler C.M. (2024). Emicizumab: the hemophilia A game-changer. Haematologica.

[6] Kitazawa T., Shima M. (2020). Emicizumab, a humanized bispecific antibody to coagulation factors IXa and X with a factor VIIIa-cofactor activity. Int J Hematol.

[7] Lauritzen B., Bjelke M., Björkdahl O., Bloem E., Keane K., Kjalke M. (2022). A novel next-generation FVIIIa mimetic, Mim8, has a favorable safety profile and displays potent pharmacodynamic effects: results from safety studies in cynomolgus monkeys. J Thromb Haemost.

[8] Lund J., Bjelke J., Granata D., Egebjerg T., Johansson E., Zhuoran W. (2024). Novel FVIIIa-mimetic molecule with the potential to be the first oral treatment for severe hemophilia A [abstract]. Res Pract Thromb Haemost.

[9] Teranishi-Ikawa Y., Soeda T., Koga H., Yamaguchi K., Kato K., Esaki K. (2024). A bispecific antibody NXT007 exerts a hemostatic activity in hemophilia A monkeys enough to keep a nonhemophilic state. J Thromb Haemost.

[10] European Medicines Agency. Hemlibra (emicizumab) (2024). Summary of product characteristics. https://www.ema.europa.eu/en/medicines/human/EPAR/hemlibra.

[11] Food and Drug Administration (2025). Hemlibra (emicizumab) labeling package insert. https://www.accessdata.fda.gov/drugsatfda_docs/label/2025/761083s020lbl.pdf.

[12] Østergaard H., Lund J., Greisen P.J., Kjellev S., Henriksen A., Lorenzen N. (2021). A factor VIIIa-mimetic bispecific antibody, Mim8, ameliorates bleeding upon severe vascular challenge in hemophilia A mice. Blood.

[13] Lentz S.R., Chowdary P., Gil L., Lopez-Jaime F.J., Mahlangu J., Matytsina I. (2024). FRONTIER1: a partially randomized phase 2 study assessing the safety, pharmacokinetics, and pharmacodynamics of Mim8, a factor VIIIa mimetic. J Thromb Haemost.

[14] Mancuso M.E., Chan A.K.C., Shanmukhaiah C., Lyu C.J., Zdziarska J., Mahlangu J. (2026). Mim8 bispecific antibody prophylaxis in hemophilia A with or without inhibitors. N Engl J Med.

[15] Persson P., Amstrup A.B., Coester H.V., Matytsina I., Bas S. (2023). Mim8, a novel factor VIIIa mimetic bispecific antibody, shows favorable safety and pharmacokinetics in healthy adults. Res Pract Thromb Haemost.

[16] Lenting P.J., Denis C.V., Christophe O.D. (2017). Emicizumab, a bispecific antibody recognizing coagulation factors IX and X: how does it actually compare to factor VIII?. Blood.

[17] Lenting P.J. (2020). Laboratory monitoring of hemophilia A treatments: new challenges. Blood Adv.

[18] Mahlangu J., Iorio A., Kenet G. (2022). Emicizumab state-of-the-art update. Haemophilia.

[19] Dargaud Y., Lienhart A., Janbain M., Le Quellec S., Enjolras N., Negrier C. (2018). Use of thrombin generation assay to personalize treatment of breakthrough bleeds in a patient with hemophilia and inhibitors receiving prophylaxis with emicizumab. Haematologica.

[20] Dargaud Y., Lienhart A., Negrier C. (2010). Prospective assessment of thrombin generation test for dose monitoring of bypassing therapy in hemophilia patients with inhibitors undergoing elective surgery. Blood.

[21] Ferrière S., Peyron I., Christophe O.D., Kawecki C., Casari C., Muczynski V. (2020). A hemophilia A mouse model for the *in vivo* assessment of emicizumab function. Blood.

[22] Kizilocak H., Marquez-Casas E., Malvar J., Carmona R., Young G. (2021). Determining the approximate factor VIII level of patients with severe haemophilia A on emicizumab using *in vivo* global haemostasis assays. Haemophilia.

[23] Shima M., Hanabusa H., Taki M., Matsushita T., Sato T., Fukutake K. (2016). Factor VIII-mimetic function of humanized bispecific antibody in hemophilia A. N Engl J Med.

[24] Duarte R.C.F., Ferreira C.N., Rios D.R.A., Reis H.J.D., Carvalho M.D.G. (2017). Thrombin generation assays for global evaluation of the hemostatic system: perspectives and limitations. Rev Bras Hematol Hemoter.

[25] Shaw J.R., James T., Douxfils J., Dargaud Y., Levy J.H., Brinkman H.J.M. (2023). Thrombin generation, bleeding and hemostasis in humans: protocol for a scoping review of the literature. PLoS One.

[26] Panova-Noeva M., van der Meijden P.E.J., Ten Cate H. (2019). Clinical applications, pitfalls, and uncertainties of thrombin generation in the presence of platelets. J Clin Med.

[27] van Meegeren M.E., Roosendaal G., Jansen N.W., Lafeber F.P., Mastbergen S.C. (2013). Blood-induced joint damage: the devastating effects of acute joint bleeds versus micro-bleeds. Cartilage.

[28] Ninivaggi M., Apitz-Castro R., Dargaud Y., de Laat B., Hemker H.C., Lindhout T. (2012). Whole-blood thrombin generation monitored with a calibrated automated thrombogram-based assay. Clin Chem.

[29] de Laat-Kremers R.M.W., Ninivaggi M., Devreese K.M.J., de Laat B. (2020). Towards standardization of thrombin generation assays: inventory of thrombin generation methods based on results of an International Society of Thrombosis and Haemostasis Scientific Standardization Committee survey. J Thromb Haemost.

[30] Valke L.L.F.G., Rijpma S., Meijer D., Schols S.E.M., van Heerde W.L. (2022). Thrombin generation assays to personalize treatment in bleeding and thrombotic diseases. Front Cardiovasc Med.

[31] Ezban M., Vad K., Kjalke M. (2014). Turoctocog alfa (NovoEight®)--from design to clinical proof of concept. Eur J Haematol.

[32] Schmitt C., Adamkewicz J.I., Xu J., Petry C., Catalani O., Young G. (2021). Pharmacokinetics and pharmacodynamics of emicizumab in persons with hemophilia A with factor VIII inhibitors: HAVEN 1 study. Thromb Haemost.

[33] Hemker H.C., Giesen P., Al Dieri R., Regnault V., de Smedt E., Wagenvoord R. (2003). Calibrated automated thrombin generation measurement in clotting plasma. Pathophysiol Haemost Thromb.

[34] Lund J., Jensen K., Burnier L., Ezban M. (2023). *In vitro* effects of combining Mim8 with factor VIII, FVIIa, and activated prothrombin complex concentrates in thrombin generation assays. J Thromb Haemost.

[35] Ay Y., Balkan C., Karapinar D.Y., Akin M., Bilenoğlu B., Kavakli K. (2012). Feasibility of using thrombin generation assay (TGA) for monitoring of haemostasis during supplementation therapy in haemophilic patients without inhibitors. Haemophilia.

[36] Chen P., Jani J., Streiff M.B., Zheng G., Kickler T.S. (2019). Evaluation of global hemostatic assays in response to factor VIII inhibitors. Clin Appl Thromb Hemost.

[37] Rezigue H., Josset L., Nougier C., Lund J., Dargaud G.Y. (2025). *In vitro* effects of Mim8 and combined Mim8-bypassing therapy on thrombin generation, thromboelastography and fibrin clot ultrastructure. Thromb Res.

[38] Appel I.M., Grimminck B., Geerts J., Stigter R., Cnossen M.H., Beishuizen A. (2012). Age dependency of coagulation parameters during childhood and puberty. J Thromb Haemost.

[39] Young G., Pipe S.W., Kenet G., Oldenburg J., Safavi M., Czirok T. (2024). Emicizumab is well tolerated and effective in people with congenital hemophilia A regardless of age, severity of disease, or inhibitor status: a scoping review. Res Pract Thromb Haemost.

[40] Pike G.N., Cumming A.M., Hay C.R., Bolton-Maggs P.H., Burthem J. (2015). Sample conditions determine the ability of thrombin generation parameters to identify bleeding phenotype in FXI deficiency. Blood.

[41] Scridon A. (2022). Platelets and their role in hemostasis and thrombosis - from physiology to pathophysiology and therapeutic implications. Int J Mol Sci.

[42] Leksa N.C., Aleman M.M., Goodman A.G., Rabinovich D., Peters R., Salas J. (2019). Intrinsic differences between FVIIIa mimetic bispecific antibodies and FVIII prevent assignment of FVIII-equivalence. J Thromb Haemost.

