# Supplementary material for: Factor VIII in vitro bioequivalence of denecimig (Mim8) hemostatic effect by thrombin generation assays
Source: Res Pract Thromb Haemost. 2026 Jan 2;10(1):103333. doi: 10.1016/j.rpth.2025.103333 (PMC12907708; doi:10.1016/j.rpth.2025.103333)
Supplement: Supplementary Material [file mmc1.docx]

# Supplementary material

Factor VIII *in vitro* bioequivalence of denecimig (Mim8) haemostatic effect by thrombin generation assays

Jacob Lund, Mirella Ezban, Kasper Jensen, David Lillicrap

**Supplementary Figure**


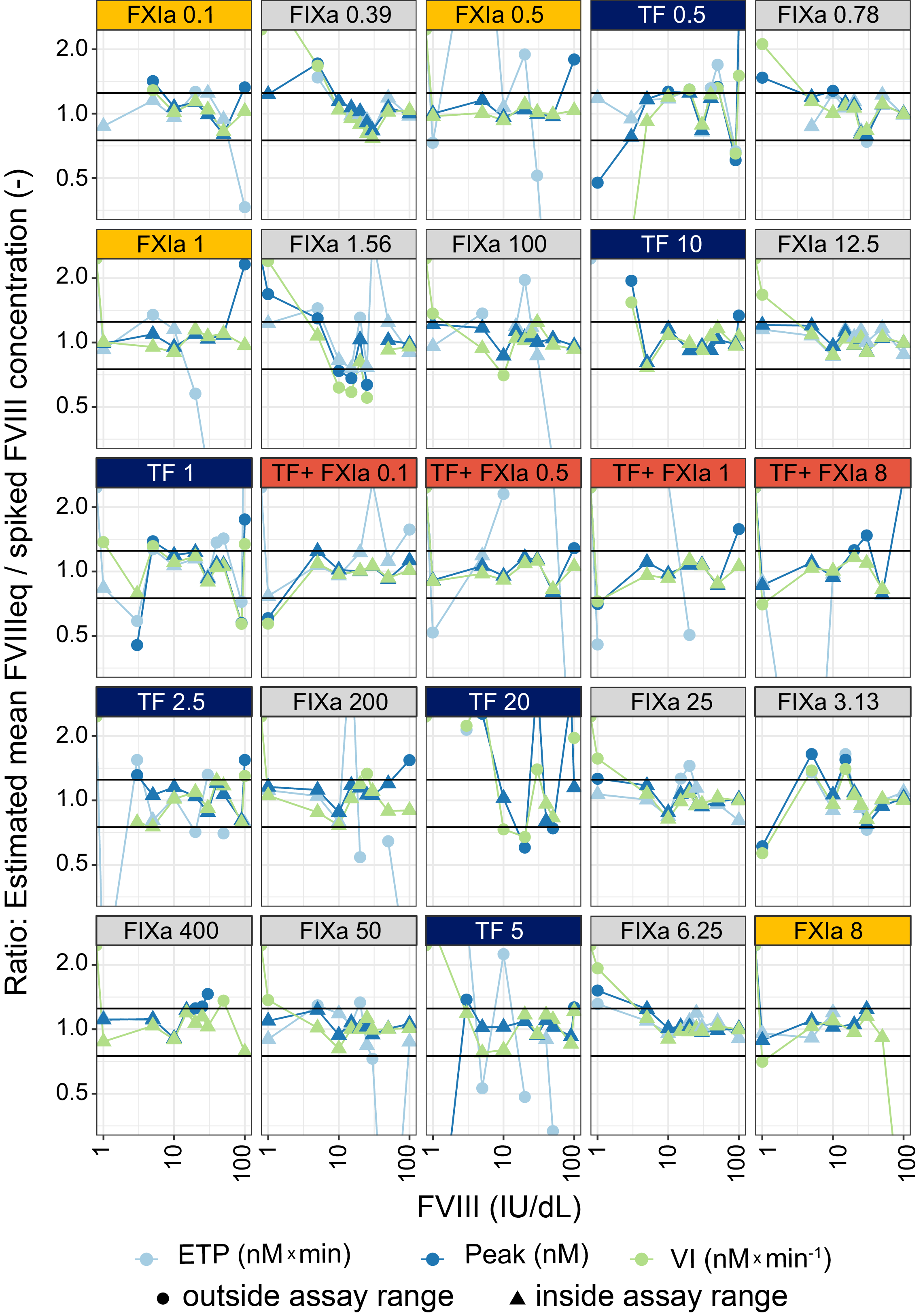


**Supplementary Figure:** Assay range for FVIII activity recovery defined as a ratio between estimated FVIII bioequivalent activity to the concentration of spiked FVIII between 0.75 and 1.25. Each facet is a thrombin generation trigger condition with header coloured according to trigger type (TF: Blue; FXIa: Yellow; FXIa added 1 pM TF: Red, FIXa: Grey) and concentration in its relevant unit (TF and FIXa: nM; FXIa and FXIa added 1 pM TF: mU/mL). Triangles denote the ratio between 0.75 and 1.25 (i.e. successful FVIII activity recovery), while circles denote values <0.75 or >1.25. ETP, endogenous thrombin potential; FVIII, factor VIII; FVIIIeq, factor VIII equivalence, activated factor XI; TF, tissue factor; VI, velocity index.

Supplementary Table

|  |  |  | **Average FVIII bioequivalence ± SD (n of experimental repeats)** | |
| --- | --- | --- | --- | --- |
| **TGA parameter** | **Trigger  condition** | **Assay range  for FVIII bioequivalence** | **50 µg/mL Emi-SIA** | **5 µg/mL denecimig** |
| **ETP  (nM × min)** | 0.39 pM FIXa | 10–100 IU/dL | 66.94 ± 9.38 (3) | NA |
|  | 0.78 pM FIXa | 5–100 IU/dL | 26.46 ± 29.4 (3) | NA |
|  | 1.56 pM FIXa | 1–100 IU/dL | 40.7 ± 44.72 (3) | NA |
|  | 3.13 pM FIXa | 10–100 IU/dL | 68.71 ± 32.22 (3) | NA |
|  | 6.25 pM FIXa | 5–100 IU/dL | 35.97 ± 2.81 (3) | 98.89 ± 8.99 (3) |
|  | 12.5 pM FIXa | 1–100 IU/dL | 26.26 ± 3.61 (3) | 67.25 ± 20.68 (3) |
|  | 25 pM FIXa | 1–100 IU/dL | 53.2 ± 16.82 (3) | 73.04 ± 31.78 (3) |
|  | 50 pM FIXa | 1–100 IU/dL | 18.76 ± 7.37 (3) | 64.84 ± 63.68 (3) |
|  | 400 pM FIXa | 1–5 IU/dL | 3.71 ± 11.82 (3) | NA |
|  | 0.1 mU/mL FXIa | 1–50 IU/dL | 24.35 ± 16.67 (7) | NA |
|  | 8 mU/mL FXIa | 1–10 IU/dL | NA | 5.79 ± 7.19 (7) |
|  | 0.5 pM TF | 1–30 IU/dL | 25.2 ± 9.24 (6) | NA |
|  | 2.5 pM TF | 5–100 IU/dL | 53.33 ± 32.91 (6) | NA |
|  | 1 pM TF + 0.1 mU/mL FXIa | 1–50 IU/dL | 26.23 ± 15.3 (7) | NA |
|  | 1 pM TF + 8 mU/mL FXIa | 1–10 IU/dL | 1.79 ± 9.72 (7) | NA |
| **Peak**  **(nM)** | 0.39 pM FIXa | 1–100 IU/dL | 16.19 ± 3.44 (3) | 41.54 ± 9.99 (3) |
|  | 0.78 pM FIXa | 5–100 IU/dL | 10.97 ± 1.23 (3) | 43.63 ± 1.62 (3) |
|  | 1.56 pM FIXa | 20–100 IU/dL | NA | 24.47 ± 2.68 (3) |
|  | 3.13 pM FIXa | 10–100 IU/dL | 11.7 ± 1.64 (3) | 37.63 ± 6.94 (3) |
|  | 6.25 pM FIXa | 5–100 IU/dL | 10.67 ± 0.19 (3) | 27 ± 2.13 (3) |
|  | 12.5 pM FIXa | 1–100 IU/dL | 11.47 ± 0.67 (3) | 21.31 ± 2.09 (3) |
|  | 25 pM FIXa | 5–100 IU/dL | 12.24 ± 0.83 (3) | 17.95 ± 0.8 (3) |
|  | 50 pM FIXa | 1–100 IU/dL | 8.98 ± 0.16 (3) | 13.85 ± 1.87 (3) |
|  | 100 pM FIXa | 1–100 IU/dL | 8.64 ± 0.65 (3) | 11.34 ± 0.77 (3) |
|  | 200 pM FIXa | 1–50 IU/dL | 8.45 ± 1.6 (3) | 11 ± 1.48 (3) |
|  | 400 pM FIXa | 1–15 IU/dL | 7.99 ± 1.65 (3) | 9.71 ± 1.52 (3) |
|  | 0.1 mU/mL FXIa | 10–50 IU/dL | NA | 23.38 ± 6.72 (7) |
|  | 0.5 mU/mL FXIa | 1–50 IU/dL | 6.2 ± 0.99 (7) | 10.61 ± 2.37 (7) |
|  | 1.0 mU/mL FXIa | 1–50 IU/dL | 5.64 ± 1.09 (7) | 8.35 ± 1.5 (7) |
|  | 8 mU/mL FXIa | 1–30 IU/dL | 4.74 ± 1.22 (7) | 6.05 ± 1.37 (7) |
|  | 0.5 pM TF | 3–40 IU/dL | 13.1 ± 3.77 (6) | NA |
|  | 1 pM TF | 10–50 IU/dL | 14.02 ± 4.86 (13) | 41.98 ± 14.48 (13) |
|  | 2.5 pM TF | 5–90 IU/dL | 12.16 ± 1.38 (6) | 19 ± 1.64 (6) |
|  | 5 pM TF | 5–90 IU/dL | 8.61 ± 0.26 (6) | 10.41 ± 1.5 (6) |
|  | 10 pM TF | 5–90 IU/dL | 6.43 ± 2.59 (6) | 7.77 ± 1.33 (6) |
|  | 1 pM TF + 0.1 mU/mL FXIa | 5–100 IU/dL | 11.61 ± 2.91 (7) | 29.7 ± 9.36 (7) |
|  | 1 pM TF + 0.5 mU/mL FXIa | 1–50 IU/dL | 10.42 ± 2.33 (7) | 18.64 ± 4.14 (7) |
|  | 1 pM TF + 1 mU/mL FXIa | 5–50 IU/dL | 9.09 ± 2.37 (7) | 15.14 ± 3.62 (7) |
|  | 1 pM TF + 8 mU/mL FXIa | 1–50 IU/dL | 6.95 ± 1.57 (7) | 10.63 ± 2.02 (7) |
| **Velocity (nM/min)** | 0.39 pM FIXa | 10–100 IU/dL | NA | 15.55 ± 4.77 (3) |
|  | 0.78 pM FIXa | 5–100 IU/dL | NA | 17.84 ± 1.26 (3) |
|  | 1.56 pM FIXa | 5–100 IU/dL | NA | 8.54 ± 1.01 (3) |
|  | 3.13 pM FIXa | 10–100 IU/dL | NA | 16.6 ± 4.43 (3) |
|  | 6.25 pM FIXa | 5–100 IU/dL | 5.49 ± 0.01 (3) | 12.07 ± 1.11 (3) |
|  | 12.5 pM FIXa | 5–100 IU/dL | 6.37 ± 0.43 (3) | 11.01 ± 0.82 (3) |
|  | 25 pM FIXa | 5–100 IU/dL | 7.21 ± 0.66 (3) | 11.2 ± 0.52 (3) |
|  | 50 pM FIXa | 5–100 IU/dL | 5.74 ± 0.15 (3) | 9.64 ± 1.15 (3) |
|  | 100 pM FIXa | 5–100 IU/dL | 5.83 ± 0.42 (3) | 8.36 ± 0.32 (3) |
|  | 200 pM FIXa | 1–100 IU/dL | 6.31 ± 1.03 (3) | 8.42 ± 0.84 (3) |
|  | 400 pM FIXa | 1–100 IU/dL | 7.76 ± 0.72 (3) | 8.49 ± 0.55 (3) |
|  | 0.1 mU/mL FXIa | 10–100 IU/dL | NA | 13.42 ± 4.82 (7) |
|  | 0.5 mU/mL FXIa | 1–100 IU/dL | 3.84 ± 0.58 (7) | 7.27 ± 1.91 (7) |
|  | 1.0 mU/mL FXIa | 1–100 IU/dL | 3.81 ± 0.74 (7) | 5.95 ± 1.19 (7) |
|  | 8 mU/mL FXIa | 5–50 IU/dL | NA | 5.35 ± 1.42 (7) |
|  | 0.5 pM TF | 5–40 IU/dL | 6.81 ± 2.31 (6) | 29.13 ± 7.8 (6) |
|  | 1 pM TF | 3–50 IU/dL | 7.59 ± 2.77 (13) | 21.79 ± 9.24 (13) |
|  | 2.5 pM TF | 3–90 IU/dL | 4.05 ± 1.1 (6) | 9.44 ± 1.21 (6) |
|  | 5 pM TF | 3–100 IU/dL | NA | 4.32 ± 1.53 (6) |
|  | 1 pM TF + 0.1 mU/mL FXIa | 5–100 IU/dL | 6.99 ± 1.86 (7) | 18.74 ± 6.79 (7) |
|  | 1 pM TF + 0.5 mU/mL FXIa | 1–100 IU/dL | 7.35 ± 1.79 (7) | 13.9 ± 3.34 (7) |
|  | 1 pM TF + 1 mU/mL FXIa | 5–100 IU/dL | 6.96 ± 1.87 (7) | 12.44 ± 2.92 (7) |
|  | 1 pM TF + 8 mU/mL FXIa | 5–50 IU/dL | 6.64 ± 1.63 (7) | 9.68 ± 1.95 (7) |
| **ttPeak  (min)** | 0.39 pM FIXa | 5–30 IU/dL | 24.27 ± 8.55 (3) | NA |
|  | 1.56 pM FIXa | 5–25 IU/dL | 12.46 ± 12.71 (3) | 23.19 ± 8.48 (3) |
|  | 0.1 mU/mL FXIa | 5–20 IU/dL | 13.47 ± 3.08 (7) | 14.5 ± 127.57 (7) |
|  | 0.5 mU/mL FXIa | 5–50 IU/dL | 19.75 ± 5.6 (7) | NA |
|  | 1.0 mU/mL FXIa | 5–50 IU/dL | 33.23 ± 17.49 (7) | 27.93 ± 137.34 (7) |
|  | 1 pM TF + 0.1 mU/mL FXIa | 5–100 IU/dL | NA | 6.03 ± 5.95 (7) |
|  | 1 pM TF + 0.5 mU/mL FXIa | 5–50 IU/dL | NA | 11.04 ± 3.8 (7) |
|  | 1 pM TF + 1 mU/mL FXIa | 1–50 IU/dL | 5.98 ± 1.66 (7) | 13.22 ± 3.56 (7) |
|  | 1 pM TF + 8 mU/mL FXIa | 1–50 IU/dL | 8.35 ± 1.6 (7) | 23.33 ± 15.28 (7) |

Emi-SIA, emicizumab sequence-identical analogue; ETP, endogenous thrombin potential; FIXa, activated factor IX; FVIII, factor VIII; FXIa, activated factor XI; IU, international unit; mU, milliunit; NA, values outside the assay range; TF, tissue factor; TGA, thrombin generation assay; ttPeak, time to peak.
